# Impact of Surgery on Non-Functional Pancreatic Neuroendocrine Tumors ≤2 cm: Analyses With Propensity Score–Based Inverse Probability of Treatment Weighting

**DOI:** 10.3389/fsurg.2022.890564

**Published:** 2022-07-08

**Authors:** Jingyuan Ye, Hongyu Wu, Jinzheng Li, Changan Liu

**Affiliations:** Department of Hepatobiliary Surgery, The Second Affiliated Hospital of Chongqing Medical University, Chongqing, China

**Keywords:** non-founctional pancreatic neuroendocrine tumors, 2 centimeters, surgery, SEER, survival analysis

## Abstract

**Purpose:**

The impact of surgery on non-functional pancreatic neuroendocrine tumors (NF-PNETs) ≤2 cm is controversial. This study sought to demonstrate the impact of surgery on the prognosis of NF-PNETs ≤2 cm with different biological behaviors.

**Methods:**

Patients with NF-PNETs ≤2 cm from 2004 to 2015 in the Surveillance, Epidemiology, and End Results database were included in this study. An inverse probability of treatment weighting (IPTW) method was used to reduce the selection bias. Kaplan–Meier survival analysis and Cox proportional hazards regression were used to evaluate the effect of surgery on the prognosis.

**Results:**

In the IPTW-adjusted Cox proportional hazards regression analysis, surgery improved the cancer-specific survival (CSS) in the overall cohort (hazard ratio [HR], 0.187; 95% confidence interval [CI], 0.102–0.343; *p* < 0.001), patients with poorly differentiated or undifferentiated tumor grades (HR, 0.238; 95% CI, 0.105–0.64; *p* < 0.001), patients with distant metastasis (HR, 0.102; 95% CI, 0.021–0.496; *p* = 0.005), and patients with local invasion (HR, 0.059; 95% CI, 0.005–0.683; *p* = 0.002). Surgery did not improve the CSS in patients with lymph node metastasis only (HR, 0.26; 95% CI, 0.0462–1.461; *p* = 0.126) or patients with well or moderate differentiation while without distant and lymph node metastasis (HR, 0.387; 95% CI, 0.146–1.028; *p* = 0.057).

**Conclusions:**

Among patients with NF-PNETs ≤2 cm, different biological behaviors correlate with different prognostic impacts of surgery. As long as distant metastasis does not occur and the grade is well–moderately differentiated, these patients will not benefit from surgery no matter whether lymph node metastasis occurs or not. However, when local invasion appears in this group of patients, surgery should be performed. Moreover, patients with a tumor grade of poorly differentiated or undifferentiated or those with distant metastases may benefit from surgery.

## Introduction

With a continuously increasing occurrence rate over the last 20 years (1), pancreatic neuroendocrine tumors (PNETs) now account for 1% of all pancreatic neoplasms (2). This trend may be attributed to increased awareness, diagnostic techniques advancement, or other unidentified environmental factors or genetic factors (3). PNETs may be divided into functional and non-functional (NF-PNET) tumors. In total, 60%–90% of PNETs are clinically non-functional (4, 5). Traditionally considered as less biologically aggressive than pancreatic cancer, PNETs are increasingly recognized for their highly variable pathological potential (6, 7). Many PNETs are indolent with a low metastasis trend and favorable long-term prognosis. In contrast, other high-grade tumors show relentless early metastasis, making their biology more aggressive than ductal adenocarcinoma. Through the generic term “PNET,” a very heterogeneous disease has unfolded before us, which can be defined either as a pancreatic neoplasm or a carcinoma (8).

Surgical resection is the only radical way to treat PNETs. In general, functional PNETs and non-functional tumors >2 cm should be resected according to the recommendations of the National Comprehensive Cancer Network (V2.2021) (9). However, the management of NF-PNETs ≤2 cm remains controversial. Today, many centers (10, 11), as well as the European Neuroendocrine Tumor Society (ENETS) (12) recommend a surveillance strategy for such lesions given their low malignant potential, slow growth, and high incidence of postoperative mortality and morbidity. On the contrary, some studies have reported that surgical resection of NF-PNETs ≤2 is associated with a better survival rate (13). There is increasing recognition that the presence of small, high-grade tumors may result in aggressive behavior (14). In addition, there is increasing evidence that nodules, distant metastases, and recurrence may present in tumor cases that meet the preoperative criteria for benign disease (i.e., intrapancreatic tumors ≤2 cm) (15–18). Therefore, there is no consensus on the optimal management of NF-PNETs ≤2 cm nor regarding the impact of surgery on these patients.

The aim of this population-based study was to determine whether surgical treatment exerted a beneficial effect on overall survival (OS) and cancer-specific survival (CSS) of patients or not. In this study, we enrolled patients with NF-PNETs ≤2 cm with different oncological characteristics, including grade (poorly differentiated, undifferentiated or well–moderately differentiated), lymph node status (lymph node metastasis or not), distant metastasis status (distant metastasis metastasis or not), and regional extension (local invasion or not). We present the following article in accordance with the Strengthening the Reporting of Observational Studies in Epidemiology (STROBE) reporting checklist.

## Materials and Methods

### Database and Patient Identification

**Figure 1:**
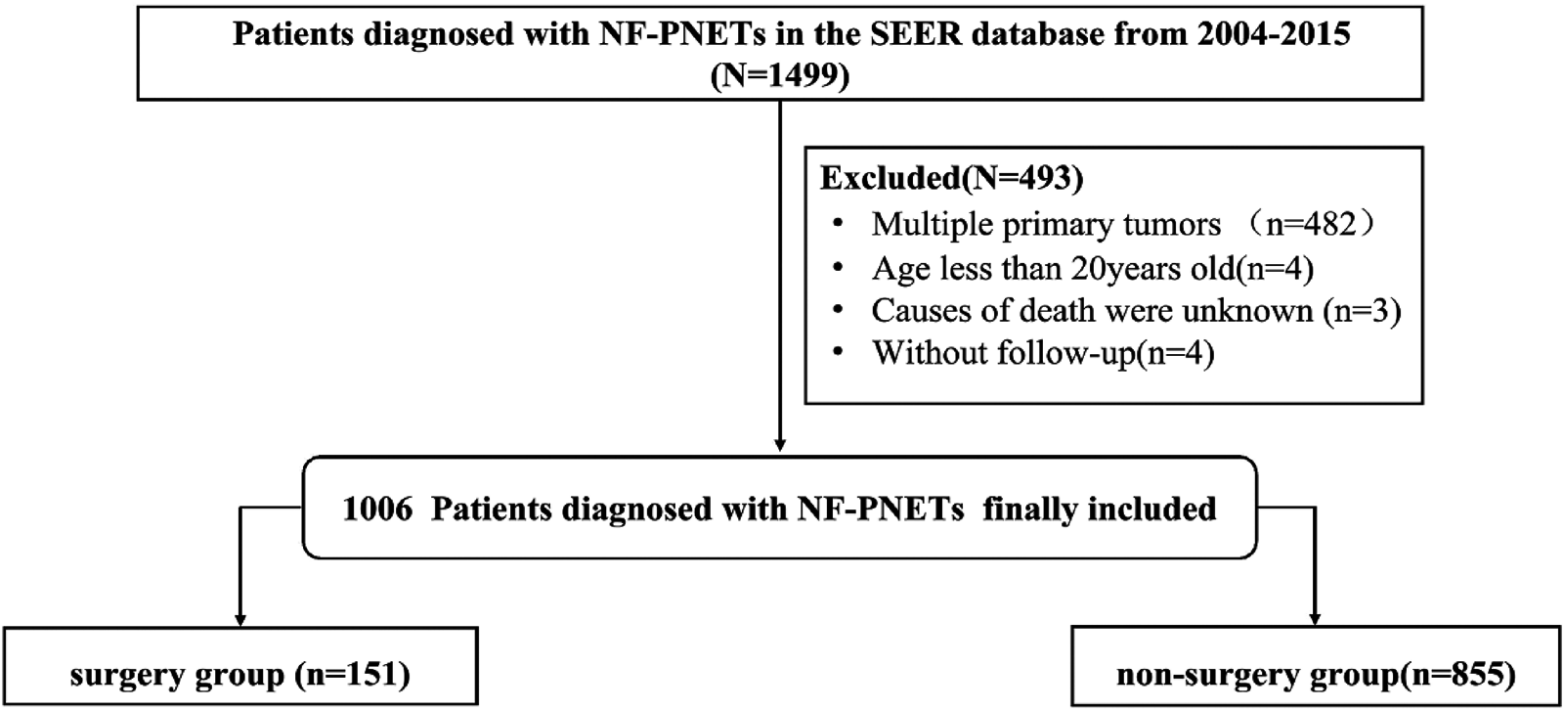
Flowchart of patient selection from the Surveillance, Epidemiology, and End Results database.

We selected potential patients who were eligible for inclusion in a retrospective cohort study from 2004 to 2015 from the Surveillance, Epidemiology, and End Results (SEER) database. The inclusion criteria for this study were as follows: (a) NF-PNETs were included on the basis of International Classification of Diseases for Oncology, third edition, (b) the tumor was ≤2 cm in size, and (c) patients had a pathological diagnosis. Patients with (a) secondary or multiple primary cancers; (b) an age of <20 years at the time of diagnosis; or (c) missing or incomplete information about survival or months of follow-up, cause of mortality, or other necessary characteristics were excluded. The patient-selected pathway is shown in Figure 1. Ethics approval and informed consent were not required for this study because the SEER study data are publicly available.

### Study Covariables and Outcomes

The variables included in this analysis were divided into 3 categories by type of information, as follows: patient-related demographics, tumor-related information, and treatment-related variables. Patient-related information included race (White, Black, or another race), diagnosed age, sex, insurance status (uninsured, insurance, or Medicaid), diagnosed year (2004–2010 or 2010–2015), and marital status (married, unmarried, divorced, separated, or widowed). Tumor-related information included tumor site (head, body, tail, and other sites), tumor size, lymph node invasion, regional extension, distant metastasis, and tumor grade (well–moderately or poorly undifferentiated). Treatment-related variables included surgery and chemotherapy.

The primary outcomes of interest were overall survival (OS) and cancer-specific survival (CSS). OS was defined as the time from the NF-PNET diagnosis date to the date of death (event occurred) or last contact (censor). CSS was defined as the time from the NF-PNET diagnosis date to the date of death due to NF-PNET (event occurred) or last contact (censor).

### Statistical Analysis

Based on the missing at random assumption approach, we used the multiple imputation method to impute missing data for race (1.69% missing), insurance (8.65% missing), marital status (5.86% missing), tumor site (9.04% missing), lymph node status (3.44% missing), and grade (20.47% missing), distant metastasis (6.26% missing), regional extension (2.68% missing), and tumor size (6.6% missing). Then, we compared the baseline characteristics before and after multiple imputation and found no significant difference (Table 1).

**Table 1:** Patient characteristics.

Characteristic	Category	Before imputation	After imputation	*p*-value
*N*		1006	1006	
Age (%)				1.000
	20–40	93 (9.24%)	93 (9.24%)	
	40–60	412 (40.95%)	412 (40.95%)	
	≥60	501 (49.80%)	501 (49.80%)	
Race (%)				0.979
	White	758 (75.35%)	768 (76.34%)	
	Black	115 (11.43%)	117 (11.63%)	
	Other	116 (11.53%)	121 (12.03%)	
	NA	17 (1.69%)	–	
Sex (%)				1.000
	Female	541 (53.78%)	541 (53.78%)	
	Male	465 (46.22%)	465 (46.22%)	
Year of diagnosis (%)				1.000
	2004–2010	233 (23.16%)	233 (23.16%)	
	2011–2015	773 (76.84%)	773 (76.84%)	
Insurance (%)				0.956
	Insured	816 (81.11%)	889 (88.37%)	
	Uninsured	18 (1.79%)	20 (1.99%)	
	Medicaid	85 (8.45%)	97 (9.64%)	
	NA	87 (8.65%)	–	
Marital status (%)				1.000
	Married	632 (62.82%)	674 (67.00%)	
	Unmarried	158 (15.72%)	168 (16.70%)	
	Divorced	88 (8.75%)	93 (9.24%)	
	Separated	10 (0.99%)	10 (0.99%)	
	Widowed	59 (5.86%)	61 (6.06%)	
	NA	59 (5.86%)	–	
Tumor site (%)				0.997
	Head	256 (25.45%)	286 (28.43%)	
	Body	219 (21.77%)	239 (23.76%)	
	Tail	338 (33.60%)	369 (36.68%)	
	Other	102 (10.14%)	112 (11.13%)	
	NA	91 (9.04%)	–	
Tumor size (median [IQR])		15.00 (10.00–18.00)	15.00 (10.00–18.00)	1.000
Grade (%)				0.094
	Well–moderate	777 (77.24%)	962 (95.63%)	
	Poorly undifferentiated	23 (2.29%)	44 (4.37%)	
	NA	206 (20.47%)	–	
Distant metastasis (%)				0.805
	Negative	845 (84.00%)	898 (89.26%)	
	Positive	98 (9.74%)	108 (10.74%)	
	NA	63 (6.26%)	–	
Regional extension (%)				0.776
	Local^a^	872 (86.68%)	892 (88.67%)	
	Extended^b^	107 (10.64%)	114 (11.33%)	
	NA	27 (2.68%)	–	
Lymph invasion (%)				0.659
	Negative	867 (86.18%)	892 (88.67%)	
	Positive	104 (10.38%)	114 (11.33%)	
	NA	35 (3.44%)	–	
Surgery (%)				1.000
	No	151 (15.01%)	151 (15.01%)	
	Yes	855 (84.99%)	855 (84.99%)	
Chemotherapy (%)				1.000
	No	954 (94.83%)	954 (94.83%)	
	Yes	52 (5.17%)	52 (5.17%)	
Cancer-specific death (%)				1.000
	No	909 (90.36%)	909 (90.36%)	
	Yes	97 (9.64%)	97 (9.64%)	
Overall survival (%)				1.000
	No	876 (87.08%)	876 (87.08%)	
	Yes	130 (12.92%)	130 (12.92%)	
Survival months (median [IQR])		36.00 (21.00–56.00)	36.00 (21.00–56.00)	1.000

*Abbreviation: IQR, interquartile range.*

^a^ *Lesions confined to the pancreas.*

^b^ *Lesions invaded the surrounding tissues of the pancreas.*

Continuous variables were described using median with interquartile range (IQR) or mean ± standard error values. Categorical variables were presented as numbers and percentages. We compared baseline characteristics between the surgery group and non-surgery group. The Wilcoxon rank-sum test was used to compare continuous variables, and categorical variables were compared using the chi-squared test. The balance in covariates was assessed using the standardized mean difference (SMD) approach. An imbalance in factors between the 2 groups was defined by an SMD of >0.1.

In non-randomized studies, the effect of treatment on outcomes can be impacted by treatment-selection bias wherein the treated cohort systematically differs from the control cohort. To account for section bias and confounding factors between the surgery group and the non-surgery group when comparing outcomes, inverse probability of treatment weighting (IPTW) was performed to balance differences in baseline demographical and clinical variables between patients who received surgery and those who did not. The Kaplan–Meier method using log-rank statistics was used to compare OS and CSS between the surgery and non-surgery groups for the IPTW-adjusted population. An IPTW-adjusted Cox proportional hazards regression analysis was performed for estimating the independent effect of surgery on the prognosis of NF-PNETs ≤2 cm.

In addition, we further conducted subgroup analyses according to grade, distant metastasis, regional extension, and lymph invasion. In each group, we also compared the OS and CSS by Kaplan–Meier analysis in the IPTW-adjusted population. An IPTW-adjusted Cox proportional hazards regression analysis was completed for each subgroup. Finally, we also conducted a sensitivity analysis for the population with missing values.

The present study conformed to the STROBE guideline (19). Statistical significance was defined by a 2-tailed *p* value of <0.05. SPSS version 24 (IBM Corporation, Armonk, NY, USA) and R version 3.6.3 (The R Foundation for Statistical Computing, Vienna, Austria; http://www.r-project.org) were used for the statistical analyses.

### Subgroup Definition

To explore the impact of surgery on the prognosis of NF-PNETs ≤2 cm with different biological behaviors, we performed subgroup analyses, comparing the OS and CSS of the surgery and non-surgery groups according to grade (G), lymph node status (N), distant metastasis status (M), and regional extension status like invasion of surrounding tissues beyond the pancreatic capsule, including blood vessels, nerves, and fat. (E). Therefore, we divided the total population into the following 5 groups: G1, M0N0G0, M0N0G0E1, M0N1G0, and M1N0G0. The G1 group included patients whose tumors appeared poorly differentiated or undifferentiated with any lymph node status, distant metastasis status, and regional extension status. The M0N0G0 group included patients whose tumors did not appear to have distant metastasis or lymph node metastasis and appeared well–moderately differentiated with any regional extension status. The M0N0G0E1 group included patients whose tumor did not appear to have distant metastasis or lymph node metastasis and appeared well–moderately differentiated with local invasion. The M0N1G0 group included patients whose tumors appeared to have lymph node metastasis, a well–moderately differentiated tumor grade, and any regional extension status without distant metastasis. Finally, the M1N0G0 group included patients whose tumors appeared to have distant metastasis (to tissue or organs, except the pancreas and lymph nodes) and appeared well–moderately differentiated without lymph node invasion.

## Results

### Baseline Characteristics in the Unadjusted and Adjusted Populations

Using the inclusion and exclusion criteria, we identified 1,006 patients in the SEER database diagnosed with NF-PNETs ≤2 cm between 2004 and 2015. Of these, there were 855 (85.0%) patients who were treated with surgery. In the overall cohort, patients without distant metastasis accounted for 89.2%, patients with tumors of a well–moderate grade accounted for 95.6%, patients with local invasion accounted for 11.3%, and patients with lymph invasion accounted for 11.3% of the population, respectively. The baseline patient, tumor, and treatment characteristics of the cohort before and after IPTW are shown in Table 2. Before IPTW adjustment, there were significant differences between the 2 groups. Most notably, compared to the non-surgery group, the surgery group contained more patients with well–moderately differentiated tumors (97.5% vs. 84.8%, *p* < 0.001), fewer patients with distant metastasis (3.9% vs. 49.7%, *p* < 0.001), more patients without chemotherapy (98.2% vs. 75.5%, *p* < 0.001), and more patients without local invasion (89.8% vs. 82.1%, *p* = 0.009). Additionally, in the surgery group, there were more insured patients (*p* = 0.036) and married patients (*p* < 0.001), which indicated a degree of selection bias in the retrospective cohort. Age, race, sex, diagnosis year, and lymph invasion were not significantly different between the 2 groups (*p* = 0.131 for age, *p* = 0.189 for race, *p* = 0.142 for sex, and *p* = 0.699 for lymph node invasion). Following IPTW adjustment, there was no significant difference between the 2 groups with SMD < 0.1 for all covariables, which indicated a favorable balance of baseline patient, tumor, and treatment characteristics between the surgery group and non-surgery group.

**Table 2:** Comparison of baseline characteristics before and after IPTW.

	Unmatched				IPTW			
Group	Level	Non-surgery	Surgery	*p*-value	Level	Non-surgery	Surgery	*p*-value
Age (%)	20–40	14 (9.3)	79 (9.2)	0.131	20–40	14.5	9.5	0.485
	40–60	51 (33.8)	361 (42.2)		40–60	37.8	41.2	
	≥60	86 (57.0)	415 (48.5)		≥60	47.7	49.3	
Race (%)	White	118 (78.1)	650 (76.0)	0.189	White	79	76.4	0.582
	Black	21 (13.9)	96 (11.2)		Black	12.5	11.3	
	Other	12 (7.9)	109 (12.7)		Other	8.5	12.2	
Sex (%)	Female	90 (59.6)	451 (52.7)	0.142	Female	51.7	53.1	0.82
	Male	61 (40.4)	404 (47.3)		Male	48.3	46.9	
Year of diagnosis (%)	2004–2010	44 (29.1)	189 (22.1)	0.074	2004–2010	21.7	23.1	0.777
	2011–2015	107 (70.9)	666 (77.9)		2011–2015	78.3	76.9	
Site (%)	Head	56 (37.1)	230 (26.9)	0.036*	Head	29.1	27.6	0.788
	Body	33 (21.9)	206 (24.1)		Body	20.7	23.5	
	Tail	43 (28.5)	326 (38.1)		Tail	35.5	37.7	
	Other	19 (12.6)	93 (10.9)		Other	14.7	11.2	
Grade (%)	Well–moderate	128 (84.8)	834 (97.5)	<0.001*	Well–moderate	95.8	96.4	0.675
	Poorly undifferentiated	23 (15.2)	21 (2.5)		Poorly undifferentiated	4.2	3.6	
Distant metastasis (%)	Negative	76 (50.3)	822 (96.1)	<0.001*	Negative	89.9	91.7	0.426
	Positive	75 (49.7)	33 (3.9)		Positive	10.1	8.3	
Chemotherapy (%)	No	114 (75.5)	840 (98.2)	<0.001*	No	94.9	96.9	0.188
	Yes	37 (24.5)	15 (1.8)		Yes	5.1	3.1	
Regional extension (%)	Local	124 (82.1)	768 (89.8)	0.009*	Local	88.3	89.1	0.855
	Extended	27 (17.9)	87 (10.2)		Extended	11.7	10.9	
Lymph invasion (%)	Negative	132 (87.4)	760 (88.9)	0.699	Negative	88.1	88.8	0.88
	Positive	19 (12.6)	95 (11.1)		Positive	11.9	11.2	
Insurance (%)	Insured	126 (83.4)	763 (89.2)	0.036*	Insured	88.3	88.9	0.284
	Uninsured	2 (1.3)	18 (2.1)		Uninsured	0.5	1.9	
	Medicaid	23 (15.2)	74 (8.7)		Medicaid	11.2	9.2	
Marital status (%)	Married	78 (51.7)	596 (69.7)	<0.001*	Married	64.2	67.7	0.942
	Unmarried	27 (17.9)	141 (16.5)		Unmarried	19	17.2	
	Divorced	25 (16.6)	68 (8.0)		Divorced	8.7	8.5	
	Seperated	3 (2.0)	7 (0.8)		Seperated	1.2	1.2	
	Widowed	18 (11.9)	43 (5.0)		Widowed	6.9	5.4	
Tumor size (mm, mean ± SD)		14.54 ± 5.02	13.56 ± 4.59	0.017*		13.78 (4.89)	13.61 (4.59)	0.778

*Abbreviations: SD, standard deviation; IPTW, inverse probability of treatment weighting.*

* *p < 0.05.*

### Surgery Group vs. Non-Surgery Group in the Overall Population

**Figure 2:**
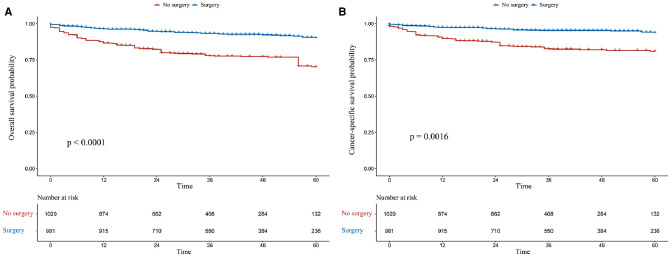
Kaplan–Meier survival estimates of overall (**A**) and cancer-specific (**B**) survival in the overall population after inverse probability of treatment weighting adjustment.

The OS rates at 1, 3, and 5 years were 95.1%, 94.4%, and 91.8% in the surgery group and 71.5%, 53.8%, and 44.0% in the non-surgery group, respectively. Meanwhile, the CSS rates at 1, 3, and 5 years were 97.8%, 96.3%, and 91.8% in the surgery group and 74.3%, 57.2%, and 49.1% in the non-surgery group. After IPTW adjustment, surgery was associated with improved OS and CSS (Figure 2). During the IPTW-adjusted Cox proportional hazards regression analysis, surgery was associated with significant OS (hazard ratio [HR], 0.205; 95% confidence interval [CI], 0.116–0.361; *p* < 0.001; Table 3) and CSS (HR, 0.187; 95% CI, 0.102­–0.343; *p* < 0.001; Table 3) benefits.

**Table 3:** Multivariable Cox proportional hazards models in the weighted population.

			Overall survival^a^			Cancer-specific survival^b^		
			HR	95% CI	*p*–value	HR	95% CI	*p*–value
Overall population	Surgery	No	1.00	–	–	1.00	–	–
		Yes	0.205	0.116–0.361	<0.001*	0.187	0.102–0.343	<0.001*
Patients with missing data	Surgery	No	1.00	–	–	1.00	–	–
		Yes	0.223	0.115–0.434	<0.001*	0.198	0.0969–0.404	<0.001*

*Abbreviation: HR, hazard ratio.*

^a^ *The OS multivariable model was constructed with surgery, age, sex, race, tumor site, tumor size, grade, distant metastasis, reginal extension, and lymph node invasion.*

^b^ *The CSS multivariable model was constructed with pre-specified variables (surgery, sex, age, grade, reginal extension and distant metastasis) to avoid overfitting considering the limited number of outcomes.*

*p < 0.05.

### Subgroup Analysis

**Figure 3:**
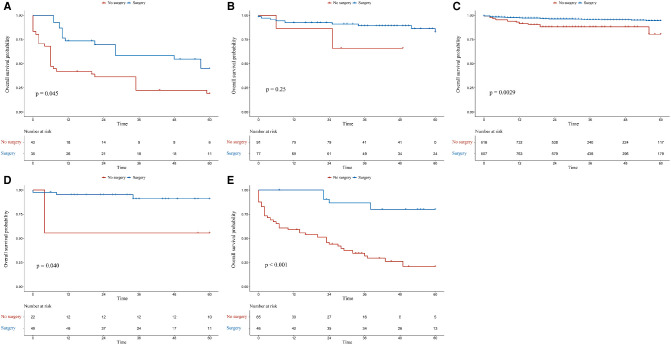
Kaplan–Meier survival estimates of overall survival in different subgroups after inverse probability of treatment weighting adjustment: (**A**) G1, (**B**) M0N1G0, (**C**) M0N0G0, (**D**) M0N0G0E1, and (**E**) M1N0G0.

**Figure 4:**
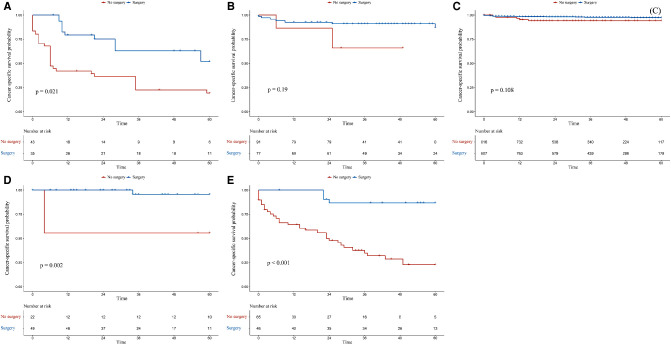
Kaplan–Meier survival estimates of cancer-specific survival in different subgroups after inverse probability of treatment weighting adjustment: (**A**) G1, (**B**) M0N1G0, (**C**) M0N0G0, (**D**) M0N0G0E1, and (**E**) M1N0G0.

In the G1 and M1N0G0 groups, patients who had undergone surgery had better OS (*p* = 0.0001 for adjusted OS of the G1 group, Figure 3A; *p* = 0.0008 for adjusted OS of the M1N0G0 group, Figure 3E) and CSS (*p* = 0.0016 for adjusted CSS of the G1 group, Figure 4A; *p* = 0.0009 for adjusted CSS of the M1N0G0 group, Figure 4E) compared to those who had not undergone surgery after IPTW adjustment.

In the M0N0G0 group, the OS of the surgery group was higher than that of the non-surgery group (*p* = 0.0029 for adjusted OS, Figure 3C), but there was no difference in CSS (*p* = 0.1082, Figure 4C). In the M0N0G0E1 group, surgery was associated with improved OS and CSS (*p* = 0.0401 for adjusted OS, Figure 3D; *p* = 0.0018 for adjusted CSS, Figure 4D). In the M0N1G0 group, there were no significant differences between adjusted CSS and OS of the 2 groups (*p* = 0.2506 for adjusted OS, Figure 3B; *p* = 0.1913 for adjusted CSS, Figure 4B).

### Independent Role of Surgery for Survival Outcomes

**Figure 5:**
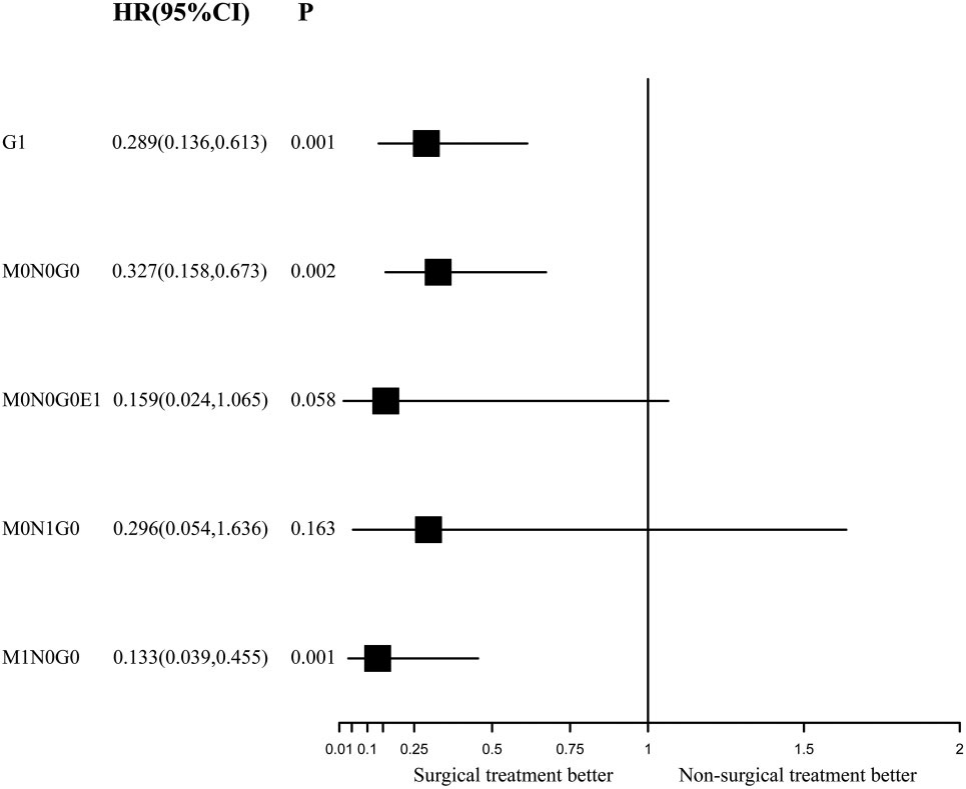
Forest plot depicting inverse probability of treatment weighting–adjusted hazard ratios of all-cause mortality in different subgroups of surgery versus non-surgery. The multivariable model was constructed with pre-specified variables (surgery, sex, age) to avoid overfitting, considering the limited number of outcomes.

**Figure 6:**
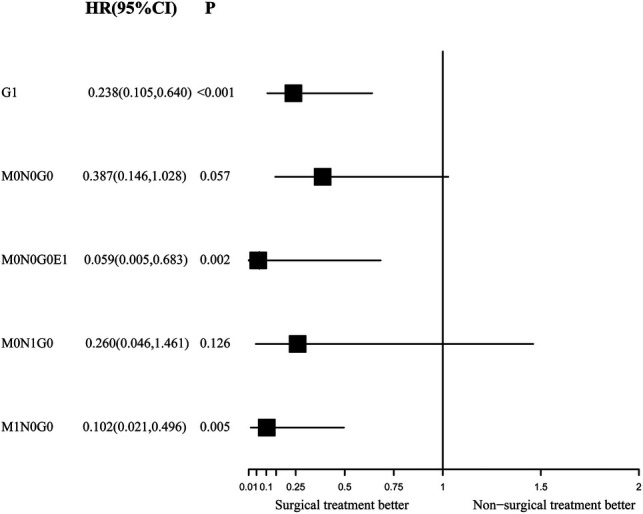
Forest plot depicting inverse probability of treatment weighting–adjusted hazard ratios of cancer-specific mortality in different subgroups of surgery versus non-surgery. The multivariable model was constructed with pre-specified variables (surgery, sex, age) to avoid overfitting, considering the limited number of outcomes.

During the IPTW-adjusted Cox proportional hazards regression analysis, we obtained almost the same results as those retrieved from the Kaplan–Meier survival analysis. Surgery was associated with significant OS and CSS benefits in the G1 group (HR of all-cause mortality, 0.289; 95% CI, 0.136–0.613; *p* = 0.001; Figure 5 and HR of cancer-specific mortality, 0.238; 95% CI, 0.105–0.64, *p* < 0.001; Figure 6) and the M1N0G0 group (HR of all-cause mortality, 0.133; 95% CI, 0.039­–0.455; *p* = 0.001; Figure 5 and HR of cancer-specific mortality, 0.102; 95% CI, 0.021–0.496; *p* = 0.005; Figure 6). The OS and CSS in the M0N1G0 group were not improved by surgery (HR of all-cause mortality, 0.296; 95% CI, 0.536  −1.636; *p* = 0.163; Figure 5 and HR of cancer-specific mortality, 0.26; 95% CI, 0.0462–1.461; *p* = 0.126; Figure 6). Surgery was associated with superior CSS in the M0N0G0E1 group (HR, 0.059; 95% CI, 0.005–0.683; *p* = 0.002; Figure 6), while there was no significant OS benefit related to surgery in the M0N0G0 group (HR, 0.387; 95% CI, 0.146–1.028; *p* = 0.057; Figure 6).

### Sensitivity Analysis

Since we performed multiple imputation on the total population, we conducted a sensitivity analysis of the population with missing values, and the results showed that surgery was associated with improved OS and CSS after IPTW adjustment (*p* = 0.0295 for adjusted OS, Supplementary Figure S1A; *p* = 0.0475 for adjusted CSS, Supplementary Figure S1B). The IPTW-adjusted Cox proportional hazards regression analysis showed that surgery had significant benefits on OS (HR, 0.223; 95% CI, 0.115–0.434; *p* < 0.001; Table 3) and CSS (HR, 0.198; 95% CI, 0.097–0.404; *p* < 0.001; Table 3).

## Discussion

With the widespread use of high-quality, cross-sectional imaging, NF-PNETs ≤2 cm are now increasingly identifiable (20). However, the optimal management strategy for patients with such tumors remains controversial. In this study, it was proved that surgery benefits the CSS of the overall population and G1, M1N0G0, and M0N0G0E1 groups, while patients in the M0N0G0E0 and M0N1G0 groups do not gain such a benefit. In a further step, we used multivariate Cox models to find that surgical treatment is an independent prognostic factor of CSS in the overall population and G1, M0N0G0E1, and M1N0G0 groups. Because of the heterogeneity of oncologic behavior and prognosis of PENTs, we should be prudent in discerning the impact of surgery on the overall population, and we believe it is reasonable and necessary to explore the prognostic impact of surgery in PNETs with different biological behaviors.

To the best of our knowledge, the present study is the first to provide compelling evidence to support different impacts of surgical treatment on patients with NF-PNETs ≤2 cm in different subgroups, including those divided according to M status, N status, regional extension, and grade, at the same time. A striking conclusion was obtained in this study by applying multiple imputation to missing data, which reduced the estimation and improved the validity. Moreover, in order to reduce the selection bias or another bias caused by the limitations of real-world research, IPTW was used to weigh the population in the adjusted analysis, which is potentially a more beneficial approach than using common matching techniques, such as retaining all the samples. After learning from prior proposals about conducting survival analysis, the influence of treatment effects on survival was analyzed in the present study, and CSS and OS were used to mitigate the unmeasured selection bias of the treatment effect.

Characterized by their indolent course and lacking early symptoms, the management of NF-PNETs ≤2 cm is still considered to be controversial. The guidelines or expert consensuses that have been published in different regions are not consistent at present. For example, the ENETS guidelines (12) suggest that observation is a reasonable option for patients with NF-PNETs ≤2 cm. However, other studies have pointed out that, even when the tumors are small (≤2 cm), they showed signs of malignant behavior, such as extra-pancreatic extension, lymph nodal metastasis, distant-organ metastasis, and recurrence, which may lead to disease-related death (21). A consensus statement announced by the Chinese Study Group for Neuroendocrine Tumors (CSNET) (22) suggested that a more aggressive approach be undertaken, except in some selected cases of NF-PNETs <1 cm or patients with incidentally discovered and unacceptable surgical risks. Patients with NF-PNETs ≤2 cm should be treated with tumor resection and careful postoperative surveillance. Meanwhile, it was suggested in the North American Neuroendocrine Tumor Society (NANETS) guidelines (23) that observation is an optimal choice for NF-PNETs ≤1 cm, while the management of NF-PNETs 1–2 cm should be considered on an individual basis according to some factors, including patient age, comorbidities, endoscopic ultrasonography–fine-needle aspiration or endoscopic ultrasonography–biopsy findings (grade, Ki-67), tumor growth status, anatomical location, extent of procedure required for complete resection, patient preferences, and access to long-term follow-up. In a meta-analysis of 714 patients with NF-PNETs <2 cm, it was discovered that PNET excision was linked to better 1-, 3-, and 5-year OS rates (13). The conclusion that surgery improves patient survival was then pushed through the literature as proof of its superiority. Therefore, whether patients with NF-PENTs ≤2 cm ought to be treated with surgery should be further explored by more accurate and specific grouping of this population. Our research has solved some of the differences in this area.

Consistent with another study (24) that evaluated 709 patients who had stage I disease according to the eighth edition of the American Joint Committee on Cancer criteria, our subgroup analysis of the M0N0G0 group found that, while surgical resection was associated with improved OS, there was no benefit offered to CSS. Compared to this earlier study, our investigation further confirmed that surgical treatment did not improve the OS or CSS of M0N1G0 patients, and this result may be related to the unclear risk-stratification of lymph node metastasis and the inconsistent impact of lymph node metastasis on survival among patients with NF-PENTs ≤2 cm (25–28). Another study using the SEER database analyzed 2,158 patients with a median tumor size of 5 cm and found that lymph node status was not a predictor of overall survival (29). These findings reinforced the observation that close follow-up is a reasonable strategy for M0N0G0 and M0N1G0 patients with NF-PNETs ≤2 cm. In addition, our study conducted a subgroup analysis of the population with local invasion (M0G0N0E1 group), where CSS was significantly improved by surgical treatment. This result may be related to the higher degree of malignancy of NF-PENTs invading the surrounding tissues of the pancreas. In addition, regional extension is related to the tumor growth rate and its anatomical location, which can be monitored by imaging methods like magnetic resonance imaging/computed tomography (30). A retrospective multi-institutional analysis that included 119 cases with confirmed NF-PNETs and resection suggested that vascular/perineural invasion is a significant prognostic factor of recurrence (31).

In order to explore the role of surgical treatment in a population with different tumor grades, a study including 380 patients (32) reported an interesting conclusion that surgical resection provides a survival benefit for patients with NF-PNETs ≤2 cm, even those with favorable well-differentiated and moderately differentiated histologies, and tumor size and margin status were not predictors of survival. Meanwhile, other studies have pointed out that poor pathological grades are signs of malignant behavior and commonly considered to be risk factors affecting the prognosis of NF-PNETs ≤2 cm (15, 33, 34). We support the conclusion that patients with poorly differentiated or undifferentiated tumors could benefit from surgical treatment, regardless of distant metastasis, lymph node metastasis, or regional extension. However, we believe that the conclusion that patients with favorable well-differentiated tumors could benefit from surgical treatment should be considered on a case-by-case basis. As mentioned earlier, we found that surgical treatment could not provide survival benefits to patients with well-differentiated tumors (those in the M0G0N0E0 and M0N1G0 groups) so long as their tumors did not appear to have regional extension or distant metastasis.

In addition, many studies have pointed out that distant metastasis is associated with the poor prognosis of NF-PNETs ≤2 cm (35–37), and we suggested that M1N0G0 patients should undergo surgery to obtain survival benefits unlike patients with pancreatic cancer, even if the grade is well-differentiated. Ye et al. (38) evaluated 758 NF-PNET patients with distant metastasis and reported that the median OS of patients treated with surgery was noticeably higher than that of those who were not treated with surgery (79 vs. 24 months). The median CSS of patients who underwent surgical resection of the primary tumor was 81 months, while that of patients who did not undergo surgical resection of the primary tumor was 26 months. However, this study included 627 NF-PNETs > 2 cm, and the NF-PNET ≤2 cm cohort was not analyzed separately from this group, so the study conclusions may be driven by these larger tumors. Therefore, our research is more convincing in terms of providing accurate findings for patients with NF-PNETs ≤2 cm with distant metastasis.

The current study has several limitations. First, given its retrospective nature, the present study has some inherent selection bias. Although the measured confounders that may influence the treatment choice and final treatment effect of patients were well balanced, any remaining unbalanced and unmeasured confounders would still bring about some bias. Second, the variables used for the survival analysis and multivariate Cox regression analysis were only a subset of the clinical and pathological features. Some important tumor markers, such as the Ki-67 index, and some positive prognostic variables, such as surgical margin status, were not available in the SEER datasets. Third, subgroup analysis was not performed for tumors ≤1 cm or 1–2 cm, and some studies have suggested these values are more rational tumor size cutoffs to identify malignancy (39–41). However, this discrepancy does not completely limit the unique effect of surgical resection of NF-PNETs ≤2 cm that we report here.

In conclusion, we have demonstrated that, first, as long as there is no distant metastasis and the tumor is well–moderately differentiated, whether lymph node metastasis occurs or not, patients may not benefit from surgery. However, if this population shows local invasion, surgery should be performed. Second, surgery should be performed if the grade is poorly undifferentiated, regardless of distant metastasis, lymph node metastasis, or regional extension. At last, patients with distant metastasis, regardless of their tumor grade, can benefit from surgery. These findings could help clinicians make better decisions about whether to choose surgery for patients with NF-PNETs ≤2 cm.

## Data Availability

Publicly available datasets were analyzed in this study. This data can be found here: https://seer.cancer.gov.

## References

[1] Yao JC Hassan M Phan A Dagohoy C Leary C Mares JE One hundred years after “carcinoid": epidemiology of and prognostic factors for neuroendocrine tumors in 35,825 cases in the United States. J Clin Oncol. (2008) 26:3063–72. 10.1200/jco.2007.15.4377 18565894

[2] Yao JC Eisner MP Leary C Dagohoy C Phan A Rashid A Population-based study of islet cell carcinoma. Ann Surg Oncol. (2007) 14:3492–500. 10.1245/s10434-007-9566-6 17896148 PMC2077912

[3] Chan JA Kulke MH. Medical management of pancreatic neuroendocrine tumors: current and future therapy. Surg Oncol Clin N Am. (2016) 25:423–37. 10.1016/j.soc.2015.11.009 27013373

[4] Metz DC Jensen RT. Gastrointestinal neuroendocrine tumors: pancreatic endocrine tumors. Gastroenterology. (2008) 135:1469–92. 10.1053/j.gastro.2008.05.047 18703061 PMC2612755

[5] Halfdanarson TR Rabe KG Rubin J Petersen GM. Pancreatic neuroendocrine tumors (PNETs): incidence, prognosis and recent trend toward improved survival. Ann Oncol. (2008) 19:1727–33. 10.1093/annonc/mdn351 18515795 PMC2735065

[6] Klimstra DS Beltran H Lilenbaum R Bergsland E. The spectrum of neuroendocrine tumors: histologic classification, unique features and areas of overlap. Am Soc Clin Oncol Educ Book. (2015):92–103. 10.14694/EdBook_AM.2015.35.92 25993147

[7] Vortmeyer AO Huang S Lubensky I Zhuang Z. Non-islet origin of pancreatic islet cell tumors. J Clin Endocrinol Metab. (2004) 89:1934–8. 10.1210/jc.2003-031575 15070966

[8] Hain E Sindayigaya R Fawaz J Gharios J Bouteloup G Soyer P Surgical management of pancreatic neuroendocrine tumors: an introduction. Expert Rev Anticancer Ther. (2019) 19:1089–100. 10.1080/14737140.2019.1703677 31825691

[9] Shah MH Goldner WS Benson AB Bergsland E Blaszkowsky LS Brock P Neuroendocrine and adrenal tumors, version 2.2021, NCCN clinical practice guidelines in oncology. J Natl Compr Canc Netw. (2021) 19:839–68. 10.6004/jnccn.2021.0032 34340212

[10] Sallinen VJ Le Large TYS Tieftrunk E Galeev S Kovalenko Z Haugvik SP Prognosis of sporadic resected small (≤2 cm) nonfunctional pancreatic neuroendocrine tumors—a multi-institutional study. HPB (Oxford). (2018) 20:251–9. 10.1016/j.hpb.2017.08.034 28988702

[11] Sallinen V Le Large TY Galeev S Kovalenko Z Tieftrunk E Araujo R Surveillance strategy for small asymptomatic non-functional pancreatic neuroendocrine tumors—a systematic review and meta-analysis. HPB (Oxford). (2017) 19:310–20. 10.1016/j.hpb.2016.12.010 28254159

[12] Partelli S Bartsch DK Capdevila J Chen J Knigge U Niederle B ENETS Consensus guidelines for standard of care in neuroendocrine tumours: surgery for small intestinal and pancreatic neuroendocrine tumours. Neuroendocrinology. (2017) 105:255–65. 10.1159/000464292 28237989

[13] Finkelstein P Sharma R Picado O Gadde R Stuart H Ripat C Pancreatic neuroendocrine tumors (panNETs): analysis of overall survival of nonsurgical management versus surgical resection. J Gastrointest Surg. (2017) 21:855–66. 10.1007/s11605-017-3365-6 28255853

[14] Liu JB Baker MS. Surgical management of pancreatic neuroendocrine tumors. Surg Clin North Am. (2016) 96:1447–68. 10.1016/j.suc.2016.07.002 27865287

[15] Kuo EJ Salem RR. Population-level analysis of pancreatic neuroendocrine tumors 2 cm or less in size. Ann Surg Oncol. (2013) 20:2815–21. 10.1245/s10434-013-3005-7 23771245

[16] Cherenfant J Stocker SJ Gage MK Du H Thurow TA Odeleye M Predicting aggressive behavior in nonfunctioning pancreatic neuroendocrine tumors. Surgery. (2013) 154:785–91; discussion 91–3. 10.1016/j.surg.2013.07.004 24074416

[17] Haynes AB Deshpande V Ingkakul T Vagefi PA Szymonifka J Thayer SP Implications of incidentally discovered, nonfunctioning pancreatic endocrine tumors: short-term and long-term patient outcomes. Arch Surg. (2011) 146:534–8. 10.1001/archsurg.2011.102 21576607 PMC3688044

[18] Watzka FM Laumen C Fottner C Weber MM Schad A Lang H Resection strategies for neuroendocrine pancreatic neoplasms. Langenbecks Arch Surg. (2013) 398:431–40. 10.1007/s00423-012-1024-7 23143147

[19] von Elm E Altman DG Egger M Pocock SJ Gøtzsche PC Vandenbroucke JP The Strengthening the Reporting of Observational Studies in Epidemiology (STROBE) statement: guidelines for reporting observational studies. Lancet (2007) 370:1453–7. 10.1016/S0140-6736(07)61602-X 18064739

[20] Dasari A Shen C Halperin D Zhao B Zhou S Xu Y Trends in the incidence, prevalence, and survival outcomes in patients with neuroendocrine tumors in the United States. JAMA Oncol. (2017) 3:1335–42. 10.1001/jamaoncol.2017.0589 28448665 PMC5824320

[21] Sallinen V Haglund C Seppänen H. Outcomes of resected nonfunctional pancreatic neuroendocrine tumors: do size and symptoms matter? Surgery. (2015) 158:1556–63. 10.1016/j.surg.2015.04.035 26070847

[22] Yang G Ji M Chen J Chen R Chen Y Fu D Surgery management for sporadic small (≤2 cm), non-functioning pancreatic neuroendocrine tumors: a consensus statement by the Chinese Study Group for Neuroendocrine Tumors (CSNET). Int J Oncol. (2017) 50:567–74. 10.3892/ijo.2016.3826 28035372

[23] Howe JR Merchant NB Conrad C Keutgen XM Hallet J Drebin JA The North American Neuroendocrine Tumor Society consensus paper on the surgical management of pancreatic neuroendocrine tumors. Pancreas. (2020) 49:1–33. 10.1097/mpa.0000000000001454 31856076 PMC7029300

[24] Powers BD Rothermel LD Fleming JB Strosberg JR Anaya DA. A survival analysis of patients with localized, asymptomatic pancreatic neuroendocrine tumors: no surgical survival benefit when examining appropriately selected outcomes. J Gastrointest Surg. (2020) 24:2773–9. 10.1007/s11605-019-04433-4 31754990

[25] Postlewait LM Ethun CG Baptiste GG Le N McInnis MR Cardona K Pancreatic neuroendocrine tumors: preoperative factors that predict lymph node metastases to guide operative strategy. J Surg Oncol. (2016) 114:440–5. 10.1002/jso.24338 27334541

[26] Fischer L Bergmann F Schimmack S Hinz U Prieß S Müller-Stich BP Outcome of surgery for pancreatic neuroendocrine neoplasms. Br J Surg. (2014) 101:1405–12. 10.1002/bjs.9603 25132004

[27] Brunner SM Weber F Werner JM Agha A Farkas SA Schlitt HJ Neuroendocrine tumors of the pancreas: a retrospective single-center analysis using the ENETS TNM-classification and immunohistochemical markers for risk stratification. BMC Surg. (2015) 15:49. 10.1186/s12893-015-0033-1 25928025 PMC4451725

[28] Wong J Fulp WJ Strosberg JR Kvols LK Centeno BA Hodul PJ. Predictors of lymph node metastases and impact on survival in resected pancreatic neuroendocrine tumors: a single-center experience. Am J Surg. (2014) 208:775–80. 10.1016/j.amjsurg.2014.04.003 24997491

[29] Franko J Feng W Yip L Genovese E Moser AJ. Non-functional neuroendocrine carcinoma of the pancreas: incidence, tumor biology, and outcomes in 2,158 patients. J Gastrointest Surg. (2010) 14:541–8. 10.1007/s11605-009-1115-0 19997980

[30] Sadowski SM Pieterman CRC Perrier ND Triponez F Valk GD. Prognostic factors for the outcome of nonfunctioning pancreatic neuroendocrine tumors in MEN1: a systematic review of literature. Endocr Relat Cancer. (2020) 27:R145–61. 10.1530/erc-19-0372 32229700 PMC7304521

[31] Du B Wang X Zhang W Tan Q Wei Y Shao Z. Management and outcomes of patients for non-functioning pancreatic neuroendocrine tumours: a multi-institutional analysis. ANZ J Surg. (2021). 92:787–93 10.1111/ans.17326 34723424

[32] Sharpe SM In H Winchester DJ Talamonti MS Baker MS. Surgical resection provides an overall survival benefit for patients with small pancreatic neuroendocrine tumors. J Gastrointest Surg. (2015) 19:117–23; discussion 23. 10.1007/s11605-014-2615-0 25155459

[33] Barenboim A Lahat G Nachmany I Nakache R Goykhman Y Geva R Resection versus observation of small asymptomatic nonfunctioning pancreatic neuroendocrine tumors. J Gastrointest Surg. (2020) 24:1366–74. 10.1007/s11605-019-04285-y 31197692

[34] Lombardi M De Lio N Funel N Sardella C Russo D Urbani C Prognostic factors for pancreatic neuroendocrine neoplasms (pNET) and the risk of small non-functioning pNET. J Endocrinol Invest. (2015) 38:605–13. 10.1007/s40618-014-0219-x 25501604

[35] Benetatos N Hodson J Marudanayagam R Sutcliffe RP Isaac JR Ayuk J Prognostic factors and survival after surgical resection of pancreatic neuroendocrine tumor with validation of established and modified staging systems. Hepatobiliary Pancreat Dis Int. (2018) 17:169–75. 10.1016/j.hbpd.2018.03.002 29576279

[36] Gao Y Gao H Wang G Yin L Xu W Peng Y A meta-analysis of prognostic factor of pancreatic neuroendocrine neoplasms. Sci Rep. (2018) 8:7271. 10.1038/s41598-018-24072-0 29739948 PMC5940798

[37] Jin K Luo G Xu J Zhang B Liu C Ji S Clinical outcomes and prognostic factors of resected pancreatic neuroendocrine neoplasms: a single-center experience in China. Oncol Lett. (2017) 13:3163–8. 10.3892/ol.2017.5834 28521422 PMC5431258

[38] Ye H Xu HL Shen Q Zheng Q Chen P. Palliative resection of primary tumor in metastatic nonfunctioning pancreatic neuroendocrine tumors. J Surg Res. (2019) 243:578–87. 10.1016/j.jss.2019.04.002 31031022

[39] Regenet N Carrere N Boulanger G de Calan L Humeau M Arnault V Is the 2-cm size cutoff relevant for small nonfunctioning pancreatic neuroendocrine tumors: a French multicenter study. Surgery. (2016) 159:901–7. 10.1016/j.surg.2015.10.003 26590096

[40] Zhang IY Zhao J Fernandez-Del Castillo C Braun Y Razmdjou S Warshaw AL Operative versus nonoperative management of nonfunctioning pancreatic neuroendocrine tumors. J Gastrointest Surg. (2016) 20:277–83. 10.1007/s11605-015-3043-5 26691146

[41] Dong DH Zhang XF Poultsides G Rocha F Weber S Fields R Impact of tumor size and nodal status on recurrence of nonfunctional pancreatic neuroendocrine tumors ≤2 cm after curative resection: a multi-institutional study of 392 cases. J Surg Oncol. (2019) 120:1071–9. 10.1002/jso.25716 31571225 PMC10178766

